# Exploring the Connection between Social Housing and Employment: A Scoping Review

**DOI:** 10.3390/ijerph21091217

**Published:** 2024-09-16

**Authors:** Julia Jansen-van Vuuren, Hibo Rijal, Nicole Bobbette, Rosemary Lysaght, Terry Krupa, Daniella Aguilar

**Affiliations:** 1 School of Rehabilitation Therapy, Queen’s University, Kingston, ON K7L 3N6, Canada; nicole.bobbette@queensu.ca (N.B.); lysaght@queensu.ca (R.L.); terry.krupa@queensu.ca (T.K.); d.aguilar@queensu.ca (D.A.); 2 School of Medicine, Queen’s University, Kingston, ON K7L 3N6, Canada; hrijal@qmed.ca

**Keywords:** social housing, public housing, economic development, work, employment, well-being, quality of life, low-income housing

## Abstract

Adequate housing is a social determinant of health and well-being, providing stability from which people can engage in important life activities, including self-care and productivity. Social housing is a system-level intervention that aims to provide affordable housing to people vulnerable to experiencing social and economic marginalisation. Given the importance of employment to social-economic status and overall health and well-being, we sought to better understand the available knowledge and research related to employment and living in a social housing environment. We used scoping review methodology to explore peer-reviewed research published between 2012–2022 regarding social housing and employment, identifying 29 relevant articles. Using the Psychology of Working Theory and neighbourhood effects as interpretive theoretical frameworks, we analysed the extracted data. Overall, the results affirmed that social housing residents have low employment rates conceptualised as related to the complex interplay of a range of personal and environmental factors. Most published literature was quantitative and originated from the United States. Policy and research implications are discussed, including the need for more multifaceted, person-centred interventions that support employment and ultimately promote health and quality of life for social housing residents.

## 1. Introduction

Housing is considered to be a basic human right, a social determinant of health, and a requirement for overall well-being [1,2]. Without safe, affordable, and stable housing, the ability of people to maintain employment and adequately provide for themselves and their families is compromised. In high-income countries social housing is an important social safety net; however, the landscape of social housing has changed over the years and differs considerably depending on context [3,4,5,6]. While social housing was traditionally built for working families in inner-city industrial areas, now it often exclusively houses people experiencing significant disadvantages, including individuals living with poor health or disability, who are impoverished, under or unemployed, racially marginalised, or single-parent families. There is a substantial body of research related to social housing and its effects on well-being and health [7,8,9], the challenges and support needs of social housing residents [10,11], and the effects of redevelopment, such as demolishing and replacing traditional, deteriorating inner-city public housing to improve the physical, economic, and social environment of disadvantaged neighbourhoods [12,13,14].

Of particular interest in this review is exploring what research has been published related to employment for adults living in the context of social housing with a view to understanding the extent and nature of the research conducted. Certainly, the relationship between employment participation and health and well-being is complex, but overall, research supports the potential positive impact of employment on an individual’s physical and mental health, social status, economic condition, and well-being in daily life [15,16,17]. As well as providing income, employment can increase self-esteem and self-confidence, provide social interactions, a sense of purpose, and positive role modelling for children [18,19]. For most working-age adults, engaging in employment is considered a critical activity of adulthood with its associated identity, roles, responsibilities, and potential opportunities. However, earlier research has suggested that housing assistance can negatively affect employment participation [20,21] and that persons residing in social housing have lower employment rates than the general population [22,23]. Social housing has sometimes been blamed for creating disincentives to work and perpetuating unemployment. Yet is social housing itself the problem, or are there factors related to the social housing environment or characteristics of people living in social housing that contribute to low employment? Additionally, what has been proposed to address barriers to employment for this population, and have they been successful? Given the significance of both housing and employment to health and well-being, we aimed to build a profile of the literature concerning employment and social housing residents in order to guide future research on this topic and to inform public policy and practice that supports employment and overall well-being for this population.

### 1.1. What Is Social Housing?

Different terminology has been used to describe low-cost or affordable housing, including social, subsidised, public, or council housing; this can make it confusing to know whether the same construct is being discussed and is comparable across the literature [24,25]. Some authors differentiate between social and public housing, where social housing is an umbrella term for any subsidised housing initiative (private or government), whereas public housing refers exclusively to government subsidised housing. Similarly, affordable housing is usually a broader term used for owner-occupied or rental homes that are made more affordable through various government subsidies or supports (e.g., allowances, vouchers, tax relief) [24]. Fitzpatrick and Pawson [26] define social housing as “residential accommodation provided at sub-market prices by state or not-for-profit landlords and allocated according to administrative criteria rather than price” (p. 598). For this review, we will use the term ‘social housing’ as defined by Fitzpatrick and Pawson unless specifically referring to government-owned public housing.

Apart from several earlier sporadic examples, social housing first emerged in the 19th century during industrialisation of Europe and the United States (US) where urban populations grew dramatically along with housing demands. Following the first and then second World Wars, social housing developed significantly in many countries, particularly Europe, and was primarily designed to provide decent homes for working families and former servicemen [26,27]. However, broadly speaking, in the 1970s, with the rise of neoliberalism, deregulation and reduced government spending and economic influence, federal governments in Europe, the US, United Kingdom (UK), Canada and Australia began reducing support for social housing projects and relinquished responsibility to local administrators and private investors, including charities, non-profit organisations and for-profit companies [6,9,28,29,30]. Globally, social housing policy has changed considerably with decreasing public investment in housing and a movement towards housing allowances or community housing rather than social housing provision. Moreover, reduced social housing stock, changes in demographics, and housing affordability challenges, have increased residualisation where limited social housing stock has become targeted housing of ‘last resort’ for the most marginalised populations [24,26,27,31,32,33]. Interestingly, this is not always the case, as the Chinese government recently increased their supply of public housing, almost doubling the housing stock, to manage housing shortages [25,34]. Additionally, Tunstall [33] argues that between 1990–2010, social housing in the UK became increasingly de-residualised as differences between housing groups diminished; however, social housing still primarily accommodates low-income residents. In countries such as the US, Canada, and Australia, social housing is often seen as untenable and a failed social experiment, promoting stereotypes of violence, poverty, and crime because of the concentration of impoverished communities [6,32,35,36]. This has led to various policies and initiatives to improve social housing, including redevelopment and mixed-income estates; however, these efforts have had both positive and negative outcomes [12,13,14,37,38].

Unsurprisingly, social housing today looks different depending on context. For example, some countries like Canada legislate the right to housing [39]; however, this right is not necessarily enforced or protected. Municipal authorities tend to own around half of social housing stock with the remainder operated by non-profit organisations, cooperative housing associations, national governments, or for-profit companies [24]. Countries such as Austria and Denmark have large social housing stock (over 20% of total dwellings), whereas others like Colombia and Latvia have low social housing stock—less than 2% [24]. In France, social housing comprises approximately 17% of households [40], while in Australia it accounts for less than 5% of housing stock [41]. Most social housing is allocated to the most marginalised and vulnerable according to strict eligibility criteria, including income thresholds and assets, citizenship, age, tenancy history, but also other priority needs such as disability, poor physical or mental health, family violence, leaving an institution, and risk of homelessness [24,31,42,43]. However, some countries (e.g., Norway, Denmark, and Sweden) maintain a more universalist model “entitling all citizens to good quality subsidised housing with below-market rents” (p. 8, [44]), which means a broader cross-section of the population live in social housing [24].

### 1.2. Social Housing and Employment

Research suggests that overall, residents in social housing have increasingly low rates of employment, especially females [22,23,45]. However, other research shows that social housing per se has minimal employment effects and is not correlated with higher unemployment compared to similar low-income households [45,46,47,48,49]. Stereotypes of social housing residents as being disinclined to work and simply draining the system are not supported by evidence, which suggests that many people in social housing rely on wages or legitimate social support (e.g., disability or aged pensions) for basic needs and do aspire to work or obtain a better job [22,32]. A 2018 US report found that 87% of social housing households with an adult who can work (i.e., non-elderly, non-disabled) reported at least one adult working or recently working, but of those not working, 78% cited family or educational obligations, and 28% of working-age residents reported a physical limitation that inhibited employment [50]. Additionally, policies around income thresholds for social housing and other welfare benefits can discourage people from earning too much or even pursuing employment at all due to the risk of losing benefits, particularly with insecure or erratic jobs [18,19,45]. Social housing residents who are employed are often in casual, part-time, low paying jobs that are temporary, insecure, and inadequate for self-sufficiency and they face recurrent periods of unemployment or unofficial employment (cash in hand) [18,19,45,50].

Social housing may, in fact, provide the stability necessary to seek or maintain work and facilitate opportunities for employment. For example, one US study found that for low-income single mothers, housing assistance increased the probability of employment indirectly through housing stability [51]. An Australian report found that some women in social housing were provided opportunities for paid traineeships and work experience that were specifically tailored to their needs (including the provision of subsidised childcare), and others found employment from initial volunteer opportunities [19]. Social housing can provide stability to improve social networks, which can positively affect employment outcomes (e.g., receiving help from others, information about or connections to job opportunities, role modelling, skills development) [45,52]. Social networks formed in social housing can facilitate employment skill development directly (e.g., sharing work skills and experiences) and indirectly (e.g., general social skills) [52]. However, strong social networks and ‘identity through place’ (sense of attachment/belonging) can also make social housing residents reluctant to move, even for better job opportunities [45].

### 1.3. Theoretical Framework

Social housing residents often face complex and intersectional personal and environmental barriers to employment; hence our inquiry into the connection between social housing and employment is based broadly on the conceptual frameworks of the Psychology of Working Theory (PWT) [53] as well as aspects of neighbourhood effects [54,55,56]. According to the PWT, work (paid as well as unpaid or caregiving roles) is an essential part of life and overall health and well-being, fulfilling fundamental human needs of survival and power, social connection, and self-determination. Work is influenced by social, economic, political, and historical forces. The purpose of our review is rooted in PWT, recognising that, like everyone, people living in social housing could benefit from decent work, but often face significant personal and environmental barriers. PWT also provides a basis for implementing individual as well as systemic change to improve employment outcomes for marginalised individuals, and ultimately, to promote their flourishing [57].

The PWT considers the interplay between psychological and contextual factors from a social justice lens that particularly focuses on those who are more marginalised and face significant challenges to securing work—highly relevant to social housing residents. Predictors (i.e., marginalisation, economic constraints), mediators (i.e., work volition, career adaptability), and moderators (i.e., proactive personality, critical consciousness, social support, economic conditions) can all affect people’s ability to work and opportunities for decent work. For example, social housing residents often have mental and/or physical health concerns or disability that make securing or maintaining work challenging [22,42,45]. Similarly, single mothers in social housing are more likely to be disconnected from work and welfare support [58], while youth transitioning from school to work often have minimal skills for career development or lack education due to erratic schooling [45]. Lack of appropriate work or mismatching skills to available jobs also negatively affects work outcomes [45]. This is a particularly prevalent barrier for immigrants with unrecognised qualifications, excluding them from higher-skilled work and wages and compelling them to accept low-skilled, low-paid work [59]. Personal characteristics such as struggles with self-confidence or maintaining personal hygiene and disciplined routines can also hinder social housing residents from accessing or sustaining employment [52].

Our focus on the intersection between work and the social housing context led us to also draw from theory related to the impact of environmental factors. A neighbourhood effects lens considers the environmental influences on people’s behaviours and overall well-being and there is a significant body of research showing that ‘neighbourhoods do matter’ even if the specific characteristics or mechanisms of such effects are inconclusive [54,55]. Galster [56] categorises the mechanisms of neighbourhood effects into four components: social–interactive, environmental, geographical, and institutional. Social–interactive mechanisms include the influence of social processes such as social networks (interpersonal communication and resources), cohesion and control, competition, contagion (behaviours or attitudes affected by peers), socialisation (conformity to local norms), relative deprivation (comparison to better-off neighbours) and parental mediation (parents’ influence on children). For example, negative role modelling and intergenerational patterns of unemployment can reinforce attitudes and behaviours that discourage social housing residents from working [52]. Environmental mechanisms refer to exposure to violence and toxins as well as physical surroundings (e.g., decay and noise). Earlier research demonstrated how exposure to violent crime, domestic violence, and substance abuse, and living in socially vulnerable neighbourhoods, negatively affects employment outcomes [42,45,60]. The physical qualities of social housing can also influence health, social inclusion, and well-being for low-income households (often interconnected with complex socioeconomic factors), which in turn, affects employment opportunities and engagement [38,61]. Geographical mechanisms include spatial mismatch (limited access to job opportunities) and public services; hence, lack of proximity to work and unavailable transport can be barriers to employment for social housing residents [19,22,62]. Finally, institutional mechanisms involve stigmatisation, local institutional resources (e.g., access to schools, daycares, healthcare) and market actors (e.g., private markets that can encourage or discourage behaviour such as healthy eating or drugs). Studies have examined stigma experienced by people living in social housing and how it affects their ability to find and maintain employment [19,45,63], including connecting with others outside of housing to facilitate employment [52]. Unaffordable and inadequate childcare has also been found to restrict employment for social housing residents, especially for single parents [19,45].

## 2. Methods

Based on the guidelines of Arksey and O’Malley [64], Levac et al. [65], and the PRISMA framework for systematic reviews [66], we conducted a scoping review to answer the research question: *What evidence exists concerning employment and working-age adults who reside in social housing?* To identify relevant studies, we consulted several university librarians regarding appropriate databases and keywords. The authors determined the inclusion/exclusion criteria (Table 1) iteratively through ongoing discussions.

We used Fitzpatrick and Pawson’s [26] relatively broad definition of social housing (described previously); however, we excluded studies that focused primarily on housing vouchers, rental subsidies for private accommodation, and other scattered-site housing assistance, as we were interested in the neighbourhood effects of social housing residents living in proximity to one another. We searched the following databases for peer-reviewed academic literature: CINAHL, Medline, Scopus, Web of Science, Sociology@ProQuest, and Sociological Abstracts (see Table A1 in Appendix A for sample database search strategy). We also hand-searched Google Scholar and a university library database for key words and key authors in the field.

**Figure 1:**
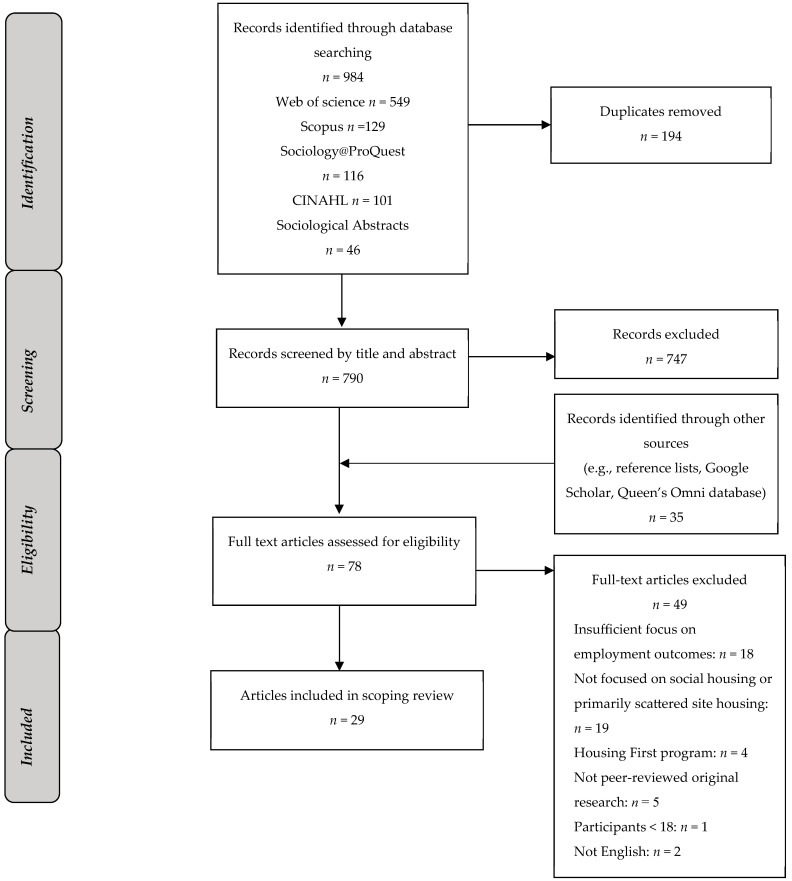
PRISMA flow diagram showing article selection process.

Using Covidence software, two authors independently screened titles and abstracts followed by full-text reviews, and a third author reviewed conflicts. The three authors met to discuss and resolve discrepancies. We screened reference lists of the included articles as well as more recent articles citing the included articles (using Google Scholar) to capture other relevant articles. Figure 1 provides a representation of article selection. We included full-text, original, peer-reviewed research articles in English between 2012 and 2022 to ensure relevance to contemporary social housing conditions.

To chart the data, we developed a Microsoft Excel spreadsheet and extracted data about general demographics for each paper, as well as information specifically relevant to our research question. Data extraction involved an iterative process where authors met to discuss the findings and ensure consistent reporting of results. We then analysed the extracted data following Hsieh and Shannon’s [67] approach to qualitatively-directed content analysis and used PWT and neighbourhood effects as interpretive theoretical frameworks to collate, summarise, and report the results.

## 3. Results

We included 29 peer-reviewed articles published between 2012 and 2022. The majority were quantitative studies (*n* = 24) and from the US (*n* = 19), although other studies were from Australia (*n* = 3), the UK (*n* = 3), Canada (*n* = 1), France (*n* = 1), India (*n* = 1) and Hong Kong (*n* = 1). Table 2 provides demographic characteristics of the articles. Looking broadly at the demographics of social housing residents in the included studies, residents often had lower education and training, lower income, were more likely to be female with children, and to identify as Black/Indigenous/racial minorities or come from a challenging past (e.g., in state care as a child, sleeping ‘rough’), had higher rates of illegal substance use, poorer mental and physical health, a higher prevalence of disability and were less likely to move out of social housing, even if a short-term disability resolved.

Overall, most of the articles confirmed earlier observations that adults living in social housing have low rates of employment and face numerous barriers to obtaining and maintaining employment. For example, one Australian study found that social housing tenants were less likely to be employed than private renters or homeowners and unemployment and living in social housing appeared to reinforce each other [68]. Similarly, a US study found that only 23% of social housing residents worked more than 15 h/week [69]. In contrast, two quantitative Australian studies did not find a statistically significant link between living in social housing and employment [70,71]. Given this lack of evidence showing negative effects of social housing on employment, Prentice and Scutella [71] suggest that providing social housing is an important safety net against homelessness without other detrimental consequences (e.g., work disincentives). Other studies also emphasised the critical positive role of social housing as a stable base from which people can access and retain employment and stabilise financially [72,73]. One mixed methods US study found that most social housing residents wanted to work and thought that residents who could work should work, but some were fearful of what would happen if they could not find work [72]. Even with employment, self-sufficiency was not guaranteed, and many social housing residents still struggled to make ends meet [72,74].

**Table 2:** Demographic information for included articles.

Author/s	Date	Title	Country	Study Design	Research Aims	Participants
Articles with description of initiative/program related to employment						
Sanbonmatsu, L., Potter, N. A., Adam, E., Duncan, G. J., Katz, L. F., Kessler, R. C., Ludwig, J., Marvakov, J., Yang, F., Congdon, W. J., Gennetian, L. A., Kling, J. R., Lindau, S. T., & McDade, T. W.	2012	The long-term effects of Moving to Opportunity on adult health and economic self-sufficiency [75].	United States	Quantitative; causal–comparative	Examine neighbourhood effects on the health and economic self-sufficiency of adults enrolled in the MTO program 10 to 15 years later.	3273 adults in public housing offered MTO voucher to relocate to an apartment (or house) in a low-poverty neighbourhood.
Ludwig, J.; Duncan, G. J.; Gennetian, L. A.; Katz, L. F.; Kessler, R. C.; Kling, J. R.; Sanbonmatsu, L.	2013	Long-term neighbourhood effects on low-income families: Evidence from Moving to Opportunity [76].	United States	Quantitative; causal–comparative	Examine the long-term effects on low-income parents and children of moving from very disadvantaged public housing to less distressed neighbourhoods.	3273 MTO adults and 5105 youth who were ages 10–20 at the end of 2007.
Barnhardt, S.; Field, E.; Pande, R.	2017	Moving to opportunity or isolation? Network effects of a randomized housing lottery in urban India [77].	India	Mixed methods; causal–comparative and qualitative interviews	Examine long-term impacts of a typical government housing program for slum dwellers.	443 participants surveyed; 21 qualitative interviews
Chyn, E.	2018	Moved to opportunity: The long-run effects of public housing demolition on children [78].	United States	Quantitative; causal–comparative	Comparison of young adult outcomes (e.g., employment, education, crime) of displaced and non-displaced children from the same public housing development to identify causal, long-term effects of moving children out of disadvantaged neighbourhoods.	Adults (>18) who had been children (age 7–18) living in Chicago Housing authority high rise public housing units—some relocated due to demolition, others remained in public housing.
Nguyen, M. T.; Rohe, W.; Frescoln, K.; Webb, M.; Donegan, M.; Han, H. S.	2016	Mobilizing social capital: Which informal and formal supports affect employment outcomes for HOPE VI residents? [69].	United States	Mixed methods; correlational and qualitative interviews	Examine the role of informal social support and formal support services for improving work outcomes among public housing residents relocated through the HOPE VI program in North Carolina.	Household heads in HOPE VI relocated to public housing in North Carolina who received case management for at least one month during the 24-month study period—data from 99 resident surveys, case management data, administrative data, and 25 interviews with case managers.
Nguyen, M. T.; Webb, M.; Rohe, W.; Noria, E.	2016	Beyond neighbourhood quality: The role of residential instability, employment access, and location affordability in shaping work outcomes for HOPE VI participants [79].	United States	Quantitative; correlational	Determine the relationship between neighbourhood quality, residential instability, employment access, location affordability, and work outcomes among people relocated as part of the HOPE VI redevelopment in Charlotte, North Carolina.	115 households of residents who could work who relocated from their original site to other public housing and who engaged with case managers for at least 5 months over the study period.
Cho, S.; Yoon, M.	2019	A study on employment of HOPE VI projects: Examining the impact of neighbourhood disadvantage, housing type diversity, and supportive services [80].	United States	Quantitative; correlational	Explore the relationship between housing type diversity, neighbourhood disadvantage and employment-related services on employment outcomes (specifically, new job placements) for HOPE VI residents.	Individuals (aged 19–64) living in 257 HOPE VI sites, both original residents (i.e., those who stayed during revitalisation or returned after revitalisation) and new residents (i.e., those who moved to the sites for the first time).
Rohe, W.; Webb, M. D.; Frescoln, K. P.	2016	Work requirements in public housing: Impacts on tenant employment and evictions [81].	United States	Mixed methods; causal–comparative and qualitative interviews	Assess the effects of work requirements on residents who could work in Charlotte Housing Authority (CHA) public housing: (a) work efforts of public housing residents; (b) rates of sanction and eviction; (c) public housing tenants’ attitudes towards work requirements.	Public housing residents who could work in work requirement sites (treatment) and non-work requirement sites (control) within CHA tenant survey data, CHA administrative data, and 43 interviews with residents in work requirement sites.
Frescoln, K.; Nguyen, M. T.; Rohe, W. M.; Webb, M. D.	2018	Work requirements and well-being in public housing [72].	United States	Mixed methods; causal–comparative and qualitative interviews	Determine the impacts on the overall well-being of public housing residents when public housing agencies implement work requirements paired with supportive services.	Adult residents able to work in Charlotte Housing Authority public housing from work requirement sites (treatment), and non-work requirement sites (control)—126 surveys of household heads and 48 interviews.
Lee, HB.; McNamara, P. E.	2018	Achieving economic self-sufficiency through housing assistance: An assessment of a self-sufficiency program of the housing authority of Champaign County, Illinois [82].	United States	Quantitative; causal–comparative	Examine the early impact of a Local Self-Sufficiency (LSS) program of the Housing Authority of Champaign County (HACC), Illinois, on participants’ household income, earnings, and employment.	Households enrolled in HACC’s LSS program, and households in a comparison non-MTW housing authority.
Treskon, M.; Gerken, M.; Galvez, M. M.	2020	Can diverse activities have a combined impact? Examining the effects of the moving to work demonstration on housing choice and self-sufficiency outcomes [83].	United States	Quantitative; causal–comparative	Explore whether Moving to Work (MTW) agencies are more effective at increasing housing choice and self-sufficiency than comparable traditional Public Housing Authorities (PHA).	Nine MTW agencies and comparison PHAs with >500 households.
Santiago, A. M., Galster, G. C.; Smith, R. J.	2017	Evaluating the impacts of an enhanced family self-sufficiency program [84].	United States	Quantitative; causal–comparative	Evaluate the impact of completing the Denver Housing Authority’s Home Ownership Program (HOP)—an enhanced FSS program.	Matched samples of between 234 and 241 cases each for both the treatment (HOP participants) and control groups.
Galster, G.C.; Santiago, A.M.; Smith, R.J.; Leroux, J.	2019	Benefit—cost analysis of an innovative program for self-sufficiency and homeownership [85].	United States	Quantitative; causal–comparative	Determine to what degree participation in Denver housing authority’s homeownership program (HOP) yielded net benefits to participants, non-participants, and society.	237 people who completed the HOP program and matched non-complier controls.
Santiago, A. M.; Leroux, J.	2022	Family self-sufficiency program outcomes for participants enrolling during and after the Great Recession [86].	United States	Quantitative; correlational	Assess whether Family Self-sufficiency (FSS) participants enrolled between 2007–2009 at the worst of the Great Recession had poorer outcomes than those who enrolled in 2010–2012 during economic recovery.	FSS participants enrolled in 2007–2009 matched to those enrolled in 2010–2012; total 424 participants.
Sha, F.; Li, B.; Guo, Y.; Law, Y. W.; Yip, P. S. F.; Zhang, Y.	2020	Effects of the Transport Support Scheme on employment and commuting patterns among public rental housing residents in Hong Kong [87].	Hong Kong	Quantitative; causal–comparative	Evaluate the effectiveness of the Transport Support Scheme (provides transport allowance to job seekers and low-income employees in remote districts) in reducing unemployment and extending the commuting distances for job opportunities for public housing residents.	Public housing residents in the labour force living in 5 districts furthest from Hong Kong’s CBD (treatment) and in districts closer to the CBD (control).
Articles highlighting discrete variables relevant to employment						
Sari, F.	2012	Analysis of neighbourhood effects and work behaviour: evidence from Paris [88].	France	Quantitative; correlational	Assess whether or not residential location influences employment probability.	46,460 working age (16–64) household heads (with a sub-sample of individuals residing in public housing) from three sub-regional administrative districts of Paris.
Feeny, S.; Ong, R.; Spong, H.; Wood, G.	2012	The impact of housing assistance on the employment outcomes of labour market programme participants in Australia [70].	Australia	Quantitative; causal–comparative	Explore whether the employment outcomes of Australians who could work in employment programs vary according to whether they receive housing assistance or not.	Working age (15–64 years) participants in compulsory employment programs (i.e., available for and seeking work), those living in government assisted housing (treatment) and those who do not (control)—data from nationally representative panel survey of Australian households between 2001–2006.
Gregoir, S.; Maury, T. P.	2013	The impact of social housing on the labour market status of the disabled [89].	United Kingdom	Quantitative; correlational	Investigate the impact of disability on housing and labour market outcomes, specifically the effect of living in social housing on labour force participation and its interaction with disability status.	Working age individuals (men aged 16–64, women aged 16–59) in English households—data from the nationally representative British Household Panel Survey from 1991–2008.
Lens, M.	2014	Employment accessibility among housing subsidy recipients [90].	United States	Quantitative; causal–comparative	Identify the extent to which housing subsidy recipients live near jobs, evaluating whether there is a spatial mismatch between these households and employment.	Comparison between subsidised housing residents and general population—data from all tracts in the 300 metropolitan statistical areas with greater than 100,000 people as of the 2000 US Census.
Distelberg, B.; Taylor, S.	2015	The roles of social support and family resilience in accessing healthcare and employment resources among families living in traditional public housing communities [91].	United States	Quantitative; correlational	Explores and differentiates the roles of community social support (internal and external) and family resilience for public housing residents regarding access and use of healthcare and employment resources.	234 families living in two of the largest public housing communities in a district of southern California.
Groenhart, L.	2015	Employment of public housing residents in Australian cities [73].	Australia	Quantitative; descriptive	Explores the current employment status of people who live in public housing in Australian cities and how this has changed over the past 30 years.	Adults living in public housing households—data from Australian Bureau of Statistics Census of Population and Housing from 1981, 1996 and 2011 for Brisbane, Sydney, Melbourne, and Adelaide.
Brucker, D. L.; Scally, C. P.	2015	Linking public housing, employment, and disability benefits for working-age people with disabilities [92].	United States	Quantitative; correlational	Explore whether levels of employment vary between people with disabilities who are living in public housing or not, and whether types of disabilities vary depending on whether they live in public housing or not.	Working-age adults (aged 25–61) with disabilities in the United States—using national population survey data.
Galster, G.; Santiago, A. M.; Lucero, J.; Cutsinger, J.	2016	Adolescent neighbourhood context and young adult economic outcomes for low-income African Americans and Latinos [93].	United States	Quantitative; causal–comparative and correlational	Determine the association between neighbourhood socioeconomic and demographic composition and employment and educational outcomes during young adulthood for African Americans and Latinos who lived in Denver Housing Authority (DHA) public housing during adolescence.	360 African American or Latino young adults (aged 18–33) who had lived in DHA public housing before age 18 for at least 2 years.
Haley, B.A.	2017	Does stigma inhibit labour force participation of young millennials who receive housing assistance? [74].	United States	Quantitative; descriptive and correlational	Provide an overview of households with young adult heads living in assisted housing, including participation in the labour force and whether stigma or alternative factors predict labour force participation.	777 US households (nationally representative sample) with heads who were 19–25 years old and living in assisted/subsidised housing.
Gregoir, S.; Maury, T. P.	2018	The negative and persistent impact of social housing on employment [68].	United Kingdom	Quantitative; correlational	Assess whether social housing contributes (and how much) to unemployment in the UK.	Working age household heads (men aged 16–64, women aged 16–59) in England; comparing social renters, private renters, and homeowners—data from the nationally representative British Household Panel Survey from 1991–2008.
Prentice, D.; Scutella, R.	2020	What are the impacts of living in social housing? New evidence from Australia [71].	Australia	Quantitative; causal–comparative	Estimate the impacts of social housing on employment, education, health, incarceration, and homelessness for Australians facing housing insecurity.	Australian individuals (>15 years) who are in social housing (treatment) and those who are potentially eligible for social housing (control)—data from 2 nationally representative household longitudinal surveys.
Jaramillo, A.; Rohe, W. M.; Webb, M. D.	2021	Predicting labour-force participation among work-able public housing residents [94].	United States	Quantitative; correlational	Examine the factors predicting participation in the labour-force (those actively looking for work) among non-employed, public housing residents who could work.	335 adults who could work (<63 years old, nondisabled) living in Charlotte Housing Authority, North Carolina.
Mendly-Zambo, Z.; Power, L.; Khan, A.; Bryant, T.; Raphael, D.	2021	Islands of isolation in a modern metropolis: Social structures and the geography of social exclusion in Toronto, Ontario, Canada [95].	Canada	Qualitative; community engagement focus groups	Community assessment of perceived service needs and day-to-day lives of residents in five social housing complexes in the inner suburbs of Etobicoke, Toronto—current needs and priorities, gaps in services and programs, barriers to accessing services, and perceived influence on policy decisions that affect neighbourhood.	First focus group: 10 adult (>18 years) women of colour, mainly immigrants. Second focus group: 10 (3 male, 7 female) high school students of colour (13–24 years). Third focus group: 12 (10 female, 2 male) university attendees/graduates, and several high school students.
Zhang, M.; Galster, G.; Manley, D.; Pryce, G.	2022	The effects of social housing regeneration schemes on employment: The case of the Glasgow Stock Transfer [96].	Scotland	Quantitative; causal–comparative	Determine whether a major regeneration scheme, transferring ownership of social housing stock from Glasgow City Council to the Glasgow Housing Association, generated more employment and, if so, for whom and how.	Working-age (women aged 16–50, men aged 16–55) adults who were social housing renters in Glasglow City and other residents in Glasgow City (treatment), and social housing renters in regions surrounding Glasglow City (control).

### 3.1. Initiatives to Support Employment Outcomes

Three articles described a US social housing revitalisation program—HOPE VI [69,79,80]. HOPE VI involved replacing poor-quality public housing units with higher quality mixed-income housing as well as providing resources and support services for affected households. Some residents returned to the redeveloped housing, others relocated to different public housing sites, while others received vouchers to relocate to private-market housing. HOPE VI more broadly aimed to improve housing, neighbourhood conditions, and quality of life for social housing residents, with employment being just one element.

Three studies evaluated the long-term benefits of the Moving to Opportunity (MTO) demonstration program in the US, which was instigated in the early 1990s, where very low-income families in traditional public housing were given housing vouchers and counselling support to move to low-poverty neighbourhoods [75,76,78]. However, despite improvements in participants’ physical and mental health, Ludwig et al. [76] and Sanbonmatsu et al. [75] found no long-term benefits on economic self-sufficiency, concluding that low employment is not a direct result of living in poor public housing environments. In contrast, Chyn [78] found employment benefits for those who had moved out of traditional public housing during their childhood compared to those who had not. In an Indian context, Barnhardt et al. [77] explored long-term outcomes of a different MTO program where female slum residents who rolled beedis (local cigarettes) entered a lottery to win the opportunity to move, with their families, to improved public housing on the city outskirts. Fourteen years later, only 34% were still living in this public housing and many had returned to the slums; there were no significant differences in labour income or hours worked between those who had won the lottery (and received access to public housing) and those who had not. While the women’s work was home-based and remained relatively unchanged, many of their husbands worked in low-skilled industries in the city-centre and moving made commuting difficult as well as hindered access for their children’s education and healthcare. The authors concluded that disrupted social networks and subsequent loss of informal social insurance meant that even substantial housing subsidies and improved housing quality was not worthwhile for participants.

Several articles explored the Moving to Work (MTW) initiative, introduced by the US government in 1996, providing flexibility to housing authorities to implement innovative programs for improving cost-effectiveness of housing programs, as well as promoting housing choice and self-sufficiency for social housing residents. Two articles specifically focused on work requirements implemented by the Charlotte Housing Authority in North Carolina as part of the MTW program [72,81]. The program provided on-site case management and support but required household heads who could work to work or engage in work-related activities (e.g., community service, education) for at least 15 h/week, or otherwise face sanctions, including losing rental subsidies or eventual eviction. Overall, the program demonstrated positive effects on increasing employment; however, the associated intensive case management support and enforcement were costly and employment improvements were inadequate for self-sufficiency as most available jobs were low paying and unstable [72,81]. In fact, Frescoln et al. [72] found that work requirements negatively affected welfare benefits (i.e., food stamps and Medicaid), decreasing food security and overall health. Additionally, the stipulated 15 h/week of mandatory work or work-related activities encouraged some residents to return to school and earn certificates that would improve employment prospects, but others felt this requirement impeded their education. Two other studies described self-sufficiency programs, also as part of the MTW initiative: Lee and McNamara [82] found substantial improvements in earnings and employment for those in the program compared to controls, whereas Treskon et al. [83] only found modest preliminary improvements in income.

Three articles explored the benefits of the Family Self-Sufficiency (FSS) program, established by the US government in 1990 to promote employment for social housing residents [84,85,86]. Though variable depending on the local housing authority, generally FSS participants voluntarily sign a 5-year contract and, with ongoing case-management support, develop an Individual Training and Services Plan and self-sufficiency goals; by the end of the program, they must be independent of welfare and working a specified number of hours. Participants are encouraged to save through an escrow account where rent increases (because of increased earnings) are deposited in a savings account, which participants can access upon successful completion of the program. Santiago and Leroux [86] compared FSS outcomes for those enrolled during the height of an economic recession in 2007–2009 and those in the post-recession recovery in 2010–2012; although those in 2007–2009 had greater employment gains, both groups had improvements in employment and earnings, demonstrating the benefits of FSS despite difficult economic circumstances. Two articles calculated the benefits of an enhanced FSS program that incorporated a home ownership program (HOP) [84,85]. Both studies demonstrated that intensive HOP participation resulted in increased earnings (through more hours worked and higher hourly wages), and a greater likelihood of a positive exit from public housing and future homeownership. Galster et al. [85] found that HOP provided significant benefits to participants, but also to non-participants and society more generally, through increased tax revenue and a reduced need for housing assistance.

One study evaluated Hong Kong’s transport subsidy scheme (TSS), which was a 2007 government initiative involving travel expense reimbursement for low-income people seeking better jobs in other districts and then short-term travel rebates to stay in these jobs [87]. The study focused on social housing residents as representative of a low-income population, finding that, overall, the TSS had positive, though small, employment effects.

### 3.2. The Psychology of Working Theory (PWT) and Employment Outcomes

Aligning with some of the predictors of securing decent work in Duffy et al.’s [53] PWT, many studies highlighted the importance of personal factors or characteristics that influenced employment outcomes for people living in social housing. In fact, Jaramillo et al. [94] concluded that individual factors (i.e., age, mental health, education, and jobs training) were more important barriers than external/environmental factors for labour force participation. However, they acknowledged that while systemic or structural barriers (i.e., car access and safety) may be less important in deciding to look for work, they may pose barriers for finding and maintaining work.

According to the PWT, characteristics such as disability, race, class, and gender contribute to social identities of marginalisation or privilege, which can predict work outcomes. In our findings, disability and poor health were consistently shown to negatively affect employment among social housing residents [68,70,74,89,92,94]. For example, Gregoir and Maury [89] found that people with disabilities were more likely to become inactive and to move into social housing than non-disabled individuals, and those who were unemployed/inactive were more likely to become disabled. The authors discussed how disability can directly affect employment due to specific performance limitations from the disability, but also indirectly through becoming a social housing tenant because of work disincentives with the potential loss of housing benefits. They found that many people stayed in social housing for prolonged periods, and even those whose disability impairments decreased over time tended to continue living in social housing. Although living in social housing may be a disincentive to work, Brucker and Scally [92] suggested that social housing alone does not provide adequate support for individuals with disabilities to find and maintain employment, and there are complex, multidimensional needs that contribute to lower employment rates.

Several of our included studies indicated that gender, age, and parental responsibilities influenced employment outcomes [74,87,94,96]; however, findings were mixed. For example, Jaramillo et al. [94] found that those who were older and without children were less likely to be working, speculating that discouragement and changing life priorities meant older adults were less inclined to seek work. Generally, women were more likely to move into social housing and be unemployed than men, particularly married men [68,89,96]. According to Zhang et al. [96], having dependent children correlated with less favourable employment outcomes, yet Gregoir and Maury [68] found that having more children increased the likelihood of homeownership. Young adult household heads with children had low household incomes, but those in the labour force were better-off than those not working; having more and younger children also decreased the likelihood of working [74] and was correlated with lower household income [82]. Conversely, Feeny et al. [70] found that children and ethnicity had no effect on employment probability for social housing residents in Australia (although this may have been due to insufficient data), but the employment probability increased up to age 24, then decreased. Gender and age also influenced the employment benefits of Hong Kong’s transport scheme (TSS). The authors found that the risk of unemployment was significantly reduced amongst males (2.9%) but not females, while cross-district employment was significantly increased amongst females (3.6%) but not males and only for those aged 45–64 for both outcomes [87]. The authors speculated that such gender differences may be because middle-aged men are often breadwinners and becoming unemployed may put more social and financial pressure on them to seek work; hence, the importance of the TSS. For women, the TSS may cause them to re-evaluate a preference for shorter commutes (due to other household/caring responsibilities) if jobs further away provide greater financial benefit. The authors also acknowledged that the TSS primarily benefitted middle-aged men who are most likely to be motivated to look for work.

As well as marginalisation, economic constraints are another predictor of securing decent work according to the PWT, including influences on early childhood development [53]. Several studies demonstrated how childhood in social housing is negatively associated with employment outcomes [78,94]. The younger their age when children relocate from social housing the better in terms of employment participation and earnings, as well as lower school dropout rates and a higher chance of attending post-secondary education [78]. Similarly, economic conditions, such as how much and what type of work is available, can moderate employment outcomes; for example, Groenhart [73] found that Australian social housing residents were overrepresented in healthcare, service industries and manufacturing jobs, which is both positive and negative, as manufacturing has declined but service industries have increased.

### 3.3. Neighbourhood Effect Mechanisms and Employment Outcomes

To categorise some of the environmental factors that were found to contribute to employment outcomes for social housing residents, we used Galster’s [56] mechanisms of neighbourhood effects.

#### 3.3.1. Social–Interactive Mechanisms

These factors include social and relational influences that can positively or negatively contribute to employment for social housing residents. For example, in a US study of young adult headed social housing households, Haley [74] found that the number of adults in the household significantly predicted labour force participation (1.9 times more likely with each additional adult). In several US studies, children provided the motivation to meet work requirements and parents wanted to be positive role models related to work [72,82]. Role models, social norms and networks were found to influence employment for social housing residents in both the US and France: Galster et al. [93] found that African-American and Latino youth in social housing who grew up in families with more resources and neighbourhoods with higher occupational prestige (i.e., more workers in professional roles as opposed to labourers) had better employment outcomes and lower welfare dependency (especially for African-Americans). Similarly, in Paris, Sari [88] showed that public housing residents in more deprived neighbourhoods with more immigrants and lower education had reduced probability of being employed. For Indian slum residents, social networks were highly valued, causing many to move out of high-quality, subsidised public housing and return to the slums [77]. According to Distelberg and Taylor [91], families in social housing with greater external community support had better access to employment resources, while increased resilience and internal community supports were associated with higher external support. However, those in social housing often had minimal external support with limited social networks and resources to access job opportunities [91,95]. Conversely, Nguyen et al. [69] found that social resources can negatively affect employment, in that close relationships with neighbours/other social housing residents can drain resources and inhibit work.

Formal social support from case managers can positively affect employment outcomes as Nguyen et al.’s [69] results showed. Enrolment in case management and positive relations with case managers had the most significant positive influence on average weekly hours worked for residents in a HOPE VI relocation program. This was due to the quality of relationships where case managers took the time to listen and build trust, allayed fears around relocation and engaging in work/education, addressed short-term crises and behavioural issues, encouraged improvement in economic situations, and provided knowledge and bridging capital (i.e., networks) to access a range of resources and overcome employment barriers [69].

#### 3.3.2. Environmental Mechanisms

Environmental mechanisms include physical surroundings and exposure to violence as well as other toxic or unhealthy pollutants. There was minimal information from our included studies related to environmental mechanisms. Jaramillo et al. [94] found that perceptions of safety for those in social housing were somewhat predictive of labour force participation. Regarding the US HOPE VI program, Cho and Yoon [80] found that housing diversity (i.e., mixed-income redevelopments with both market-rate and subsidised homes) was a predictor of new job placement, while Nguyen et al. [79] found that neighbourhood quality did not significantly predict work outcomes, but living in more costly neighbourhoods did and was correlated with greater work consistency. The authors postulated that living in more expensive neighbourhoods requires more money, which necessitates more work, or improves motivation to work to improve the overall quality of life. Social housing residents in Toronto, Canada, described poor-quality, run-down, and overcrowded housing, as well as neglected schools in need of repairs, and trauma from past violent experiences (especially for immigrants/refugees), but these environmental factors were not directly linked to employment outcomes [95].

#### 3.3.3. Geographical Mechanisms

Access to job opportunities as well as public services and facilities constitute geographical mechanisms that can contribute to employment. Several articles suggested that social housing residents in larger cities or urban centres had better employment outcomes than those in regional areas, likely because of more and varied job opportunities and better transport [70,73]. Similarly, Lens [90] found that while competition negatively affected job opportunities for low-skilled unemployed workers, when comparing job openings per labour-force member, those living in public housing were actually close to more job openings, as public housing was concentrated in areas of high employment growth. Nguyen et al. [79] also found that residents relocating to other traditional US public housing locations had better employment access compared to housing voucher holders, attributing this to the location of public housing in job-dense city centres. In contrast, voucher holders are often pushed towards cheaper housing in distant, less job-accessible locations. For Indian slum residents, while minimally affecting women with home-based work, moving to newly built public housing on the city periphery negatively affected many of the men who worked in central low-skilled industries by significantly increasing their commuting time and cost [77]. However, Groenhart [73] described the increasing spatial polarisation between advantage and disadvantage in Australian cities; social housing stock has not changed locations, and therefore, residents may have less access to shifting employment locations. This was also evident for social housing residents in Toronto, who, despite living in a major city were geographically isolated from commercial areas, with mostly temporary low-paid jobs available to them [95]. Hence, these examples demonstrate that proximity of housing to appropriate jobs is a key distinguishing factor in terms of employment outcomes.

Access to transportation also affects employment outcomes for social housing residents. Nguyen et al. [69] found that US social housing residents who worked 15 h or more per week were more likely to own a car and rely less on public transport. In Paris, having a driver’s licence was correlated with better employment outcomes, but vicinity to jobs had no significant effect; the author speculated this was likely because a substantial portion of public housing is in the central city and already close to jobs [88]. In contrast, Jaramillo et al.’s [94] results showed that access to a car was not a significant predictor of labour force participation for social housing residents. Sha et al. [87] highlighted the role of public transport access for employment in Hong Kong, finding that the TSS significantly reduced the risk of unemployment by 2% and promoted the likelihood of cross-district employment by 1.2%. These employment effects were maintained after 5 years; however, the authors acknowledged that although the transport scheme reduced commute costs, it did not improve commute time, and while it may support a more flexible and accessible workforce, the effect size was small and the program costly.

#### 3.3.4. Institutional Mechanisms

Institutional mechanisms refer to local institutional resources, private market influences, and stigmatisation that can affect job opportunities. Several studies indicated that higher educational attainment and training opportunities were associated with improved employment outcomes for people in social housing [68,74,79,80] and vice versa [94]. For example, only 45% of young adult household heads in social housing who lacked a high school diploma were participating in the labour force, compared to 72% of those with some college experience [74]. However, Feeny et al. [70] found that for Australian social housing residents, qualifications were not significant predictors (although over 50% had no post-school qualifications), but labour market history was important, as more time previously spent in paid work increased employment probability. They posited that this may be because unemployment can depreciate skills and lead to discouragement to seek future employment or employers see work history as an indicator of reliability and capability. Additionally, post-secondary education did not guarantee employment for Canadian young adults in social housing and student debt added to stress due to insufficient government student support [95].

Regarding other institutional services, Frescoln et al. [72] found that lack of affordable or available childcare negatively affected employment, especially for single mothers, while other quantitative studies did not find significant associations between childcare and employment [69,80]. One study specifically explored the role of stigma in labour force participation for young adults in social housing, finding no significant direct influence (although perhaps indirectly on health) [74]. However, a qualitative study found that Canadian young adults in social housing did face discrimination and social exclusion, including unfair targeting by police, which negatively impacted future job prospects [95]. These youth felt that without decent or influential jobs, they lacked a voice, and they requested support for empowerment, mentorship, entrepreneurial programs, and career development opportunities (e.g., volunteering, networking, and resume writing) [95].

## 4. Discussion

This review examined peer-reviewed research related to employment and social housing to better understand the extent and nature of the research in order to inform policy and practice that promotes health and well-being for this frequently marginalised population. We used scoping review methodology, systematically searching the literature to broadly summarise and disseminate the findings and to identify research gaps [64]. Our searches did not reveal a large amount of published research specifically focused on employment in the context of social housing. Additionally, the research predominantly focused on quantitative studies in the United States and most of the articles related to employment programs referred to a broader goal of self-sufficiency. Our findings from the 29 included articles indicated that overall, social housing residents have low rates of employment; however, none of the studies demonstrated a simplistic causal relationship between social housing and low employment. Instead, research indicated that social housing residents face numerous personal and environmental barriers to employment and none of the proposed programs or initiatives to date offer comprehensive solutions (discussed below in more detail). Perhaps this is unsurprising, given the complexity and multiplicity of the issues. In fact, it could be argued that decent, sustainable employment for social housing residents may be considered a ‘wicked problem’—i.e., a contemporary complex problem with multiple causes, contested understandings, and significant negative health effects, requiring collective action [97]—particularly in light of the current global housing crisis and subsequent pressure on affordable housing options.

In the absence of existing theory directly related to employment and social housing, we positioned our work in relation to broad theories relevant to employment and both individual and neighbourhood contexts—Duffy et al.’s [53] PWT and neighbourhood effect mechanisms [56]. Interestingly, few of the research publications we reviewed explicitly identified the theoretical frameworks that guided the studies. Our scoping review indicated that the relationship between social housing and employment is complex and suggests that this social issue could benefit from the development of focused, mid-range theories that link macro or social level factors relevant to social housing (such as those proposed by neighbourhood effects) to micro, or individual level factors (related to individual beliefs, agency, actions etc.). Such midrange theories would systematically and empirically advance explanatory models specific to employment in the social housing context that would be useful in the development of policy and practice initiatives. In addition, such mid-range theory could be developed deductively from the existing quantitative research. However, given the dearth of qualitative research in this area, a grounded theory approach, building theory inductively from lived experience in context, may be particularly valuable.

### 4.1. The Interplay between Individual and Environmental Factors

The PWT hypothesises that marginalisation and economic constraints are predictors for securing (or being unable to secure) decent work. Although the demographic profiles of social housing residents in our included studies indicated that many of them experience intersectional marginalisation—i.e., race, gender, age—few studies specifically explored the association between these characteristics and work outcomes for social housing residents. In fact, articles that did measure correlations between personal characteristics and employment demonstrated mixed results. In general, findings suggested that women with young children and those with disabilities had poorer work outcomes; however, there was minimal exploration into why this is the case or recommendations to support people with additional barriers. The PWT also describes mediators—work volition, career adaptability—and moderators—proactive personality, critical consciousness, social support, economic conditions—that influence decent work. While environmental factors of social support and economic conditions were mentioned, none of the articles in this review explicitly explored or discussed the influence of psychological characteristics on employment. Other researchers have tested the PWT framework with diverse populations, showing that marginalisation and economic constraints directly and indirectly affect securing decent work e.g., for women of colour [98], low income Turkish workers [99], Chinese urban workers [100] and Black workers in the US [101]; this is particularly mediated through work volition, as populations who are consistently marginalised often have limited choices and resources necessary to find and maintain decent work. However, more research is needed to understand the role of such moderators and mediators [98,99,100]. Further research, both quantitative and qualitative, could test the PWT model specifically for social housing residents and include a focus on these mediators and moderators to inform policies and practices around employment conditions and support services, particularly for more vulnerable populations within social housing (e.g., single parents, individuals with disability).

Most of the included articles focused on environmental factors contributing to work outcomes, acknowledging that many employment barriers are out of the individual’s control. While this is important given the criticisms of psychological theories that put the onus, or even fault, on the individual for unemployment, there are also limitations to emphasising environmental factors and neighbourhood effects specifically. For one thing, it is difficult to quantify, measure, and prioritise neighbourhood effects to determine which factors contribute significantly to employment outcomes [54,55,56]. Additionally, Goetz [36] argues that concentrating only on environmental challenges can lead to an assumption that simply improving the environment is the solution. In high-income contexts, such as the US, UK, Canada, and Australia, this has resulted in large and expensive programs to redevelop traditional social housing blocks (especially in valuable inner-city locations) and promote mixed-income estates. However, research has shown this has had limited success and points to the complex multifaceted barriers that social housing residents face, both personally and in their surrounding environment. For example, Chaskin and Joseph’s [102] comprehensive study on the Chicago social housing redevelopment—*Integrating the Inner City: The Promise and Perils of Mixed-Income Public Housing Transformation*—highlights that, despite some benefits to the physical environment, the project failed to meet its social and economic goals, and social housing residents largely remained marginalised, poor, and disconnected from society.

### 4.2. Interventions to Support Employment: No Simple Solutions

Despite several articles exploring programs to improve self-sufficiency (i.e., HOPE VI, MTO, MTW, FSS, and TSS), the programs were almost exclusively US-based and few focused specifically on work interventions or clearly identified what attributes led to successful employment outcomes. Several articles about the FSS program showed promising results, particularly with an enhanced home-ownership program, but employment was only one aspect of the program, not the primary focus. While findings related to various programs indicated some positive effects of relocation, housing diversity, and supportive case management, overall employment rates for social housing residents remained low. Additionally, a MTW work requirement program showed increased employment rates and wage income, but it detrimentally affected health, welfare benefits, and well-being. None of the articles discussing employment interventions separately analysed or explicitly considered the distinct needs and challenges of more vulnerable groups within social housing, such as those with disabilities or single parents.

Such self-sufficiency initiatives are not without their challenges and critiques. For example, Webb et al. [103] criticised the MTW demonstration for its lack of federal oversight, limited evaluation, and conditions on housing assistance increasing housing insecurity. Likewise, Oakley et al. [104] claimed that HOPE VI did not meet its goals primarily because of insufficient support to mitigate the numerous barriers that social housing residents face in reaching self-sufficiency. In a recent study, Jaramillo and Rohe [105] highlighted the significant health challenges that many social housing residents face, hence “employment is not a reasonable or desirable outcome for some clients, such as those in poor health” (p. 15) and health screening is essential for any self-sufficiency or employment program. Our findings indicated that employment interventions are often costly (e.g., the TSS, intensive case management in MTW work requirements); however, arguably the cost of unemployment is higher, including greater welfare dependence, increased food insecurity, generational unemployment, and other ensuing social and health costs. In fact, one study in our review specifically conducted a benefit–cost analysis of an innovative self-sufficiency home-ownership program, demonstrating the significant financial and social benefits, both to participants and society more broadly [85].

Other research has indicated that simply redeveloping or relocating social housing residents has minimal effect on employment and can even be detrimental to health and well-being [106,107]. Residents in mixed-income housing may experience less stigma, more stable, safe, and better-quality environments, but relocation does not automatically promote social and economic equality and residents can face other challenges, including higher living costs and alternative forms of stigma and exclusion [108,109,110]. Hagan et al. [111] described the disruption of home and community for US social housing residents facing redevelopment, including “traumatic loss of physical places and demolition or removal of co-constructed social spaces where residents had typically come together” (p. 532). Similarly, though not focused on employment, Jaramillo et al. [112] found that MTO housing vouchers did not necessarily improve opportunity and well-being, and in fact, as opportunity increased, people’s satisfaction slightly decreased. In contrast, Kim et al. [113] found positive and sustained improvements in opportunities for voucher recipients continuing over 10–15 years, but intensive housing counselling and support were key factors. Traditional public housing estates are not themselves the cause of low employment rates. In fact, research into China’s targeted poverty alleviation program, where poor, remote households were moved to areas of increased opportunity found that only those collectively relocated (i.e., to traditional public housing) had significant improvements in income and wages, while dispersed relocation had no significant effect [114].

One initiative specifically focused on improving employment outcomes for social housing residents was Jobs Plus, a special demonstration project launched by the US government in 1998. The initial program finished in 2003 (hence why we did not find recently published studies to include in the review) but showed promising outcomes for future scale up and implementation. Unlike other employment initiatives, in the chosen sites for Jobs Plus, every resident capable of working was targeted (although the program was voluntary) and it incorporated employment services (e.g., job search assistance, skills training, childcare, and transport), financial incentives to work (e.g., reduced rent increases with increased income), and community support (e.g., disseminating employment information through neighbours). The initial results showed significant improvements in annual earnings and employment rates for diverse residents; unfortunately, this did not result in significant improvements in broader social conditions and quality of life [115]. However, follow up reports have indicated sustained long-term gains in annual earnings [116] and more recently, additional Jobs Plus programs have been implemented with promising results, although not without challenges [117].

Countries apart from the US have various organisations, policies, and programs dedicated to improving employment outcomes for social housing residents; however, there appears to be minimal peer-reviewed research related to the implementation or effectiveness of such programs. For example, in Australia, the New South Wales government initiated a program called *Opportunity Pathways* to help social housing residents obtain and maintain employment through training, coaching/mentoring, job placements, case management and on-the-job support [118]. In the UK, *Communities that Work* is a national English social housing network, supporting employment of working-age residents [119]. Unfortunately, our searches of academic databases and journals did not reveal any original research related to these or similar programs in other countries and further research into the effectiveness of such programs in diverse contexts is necessary.

### 4.3. Recommendations and Future Directions for Supporting Employment

Although this review identified limited research focused specifically on improving employment outcomes for social housing residents, we can draw out several broad recommendations for directing policies and allocating resources to address priority needs. In an Australian report, Leishman et al. [120] conducted a rapid review to identify what models or programs have been implemented for social housing residents to improve employment access and sustainability, and what elements make these programs effective. Despite evidence gaps in long-term outcomes and particular features of successful programs, overall, they found that multifaceted support services, community engagement, addressing multiple social barriers, and timely implementation improved employment outcomes. Similarly, several reports from Employment, Housing and Social Justice institutes in the UK provide policy recommendations for improving employment opportunities for social housing residents; authors highlighted the crucial role of social housing associations/providers/landlords in supporting employment as they often have the access, relationships, and trust, as well as experience working with marginalised individuals and communities. Other recommendations included providing tailored, open-ended, one-on-one support, addressing wider issues (e.g., childcare and transport), supporting collaborative programs and coordinating partnerships between various local services, and ensuring funding commitment and continuity [121,122,123,124]. Strong leadership and commitment from housing authorities, as well as dedicated staff who provide personal, caring, on-site support, was also reinforced by the Jobs Plus program [115] and highlighted in our findings related to the HOPE VI relocation program [69]. Additionally, for future Jobs Plus programs, Verma and colleagues [117] provide several recommendations: capitalising on the place-based nature of the program with staff engaging regularly with residents and actively targeting disengaged residents with additional barriers by going to them (instead of waiting for them to enter the office), starting with entry-level job placements rather than a more ambitious career approach, ongoing support for job retention and advancement, more structured case management, capacity building of case managers and support staff, and strengthening connections with employers and other workforce agencies.

One approach that may have potential for application with residents of social housing, but not identified in the review articles, is the development of local social enterprises. Social enterprises are “organisations that engage in commercial trade for a social purpose—most often to address one or more aspects of social vulnerability—rather than for the personal financial enrichment of owners or shareholders” (p. 440) [125]. Some social housing providers are in fact social enterprises themselves; for example, *Housing Plus* in Australia that reinvests any profits back into the community [126].

Work Integration Social Enterprise (WISE) is a particular form of social enterprise that was specifically designed and implemented to improve employability and opportunities for integration for those who experience forms of labour market exclusion [127], including many of the groups represented among social housing residents. For example, WISEs have been developed to address the employment marginalisation of those with disabilities [128], serious mental illnesses [129], youth [130], individuals exiting the criminal justice system [131] and formerly homeless individuals [132]. Growing evidence suggests engagement in WISEs can have a positive impact on aspects of employment, integration, and overall well-being, such as skill development, increasing income and financial stability, reducing community stigma and developing community connections [129,132,133]. Additionally, despite the substantial cost of developing and maintaining WISEs, evaluations using the social return on investment (SROI) approach have shown promising financial benefits of WISEs (albeit with limitations to the SROI methodology) [134,135]. However, understanding of the best approaches to WISE and what elements make such initiatives successful remains unclear. Policy developments and research will be needed to explore how such social enterprise models can be developed to meet the multidimensional needs of social housing residents specifically. Furthermore, there is a collective dynamic to social enterprises whereby participants identify not only with the goals, structures and processes of the enterprise, but also connect with other participants with common needs related to employment. Practice and research will need to consider the processes that would be effective in engaging social housing residents in social enterprise initiatives and building a collective identity through entrepreneurship [136].

### 4.4. Limitations

Despite efforts to ensure a thorough and comprehensive search for relevant articles, our review has several limitations that should be considered when interpreting findings. First, we included only published peer-reviewed academic literature, excluding books, reports and other grey literature that described important initiatives and recommendations to support employment outcomes and address barriers for social housing residents. Second, we recognise that social housing varies widely across countries (including funding mechanisms, resident qualifications, and policy goals) and as such, policy and intervention strategies relevant to specific outcomes such as employment can be difficult to compare. In accordance with scoping review methodology, our search parameters were broad, and we did not specify any particular region in order to identify what research has been conducted in the field more generally, and also to ascertain whether best practices can be relevant and shared across jurisdictions. Despite this intention, the identified articles were mainly from the US, and therefore do not provide an international perspective on social housing. Characteristics of social housing residents, employment experiences and outcomes, and support needs will vary significantly, and more research is needed in diverse contexts, particularly in low-and-middle-income countries. Finally, due to the difficulties inherent in defining social housing we may have missed other important literature related to social housing and employment, despite ongoing team discussions to clarify the inclusion/exclusion criteria.

## 5. Conclusions

The overall findings of this review indicated that social housing residents demonstrate limited engagement in employment, and this appears to be largely due to a complex interplay of personal and environmental barriers. The findings revealed the relevance of both individual and environmental factors to the employment of social housing residents, underscoring the theoretical relevance of the Psychology of Working theory and neighbourhood effects perspectives in understanding this complex issue. Personal attributes that contribute to marginalisation, such as disability and gender, as well as economic constraints, were identified as negatively impacting employment outcomes for social housing residents; however, neighbourhood effects from social–interactive, environmental, geographical, and institutional mechanisms can both facilitate and thwart employment outcomes. Most articles focused on quantitative studies from the US and further qualitative research and research from a range of contexts is necessary to understand the employment needs, perspectives, and lived experience of social housing residents more broadly, as well as to build relevant theory. While understanding the challenges and barriers to employment is important, our review also highlighted the need for more published research on interventions and programs that may effectively promote employment and overall quality of life in collaboration with social housing residents themselves and other key stakeholders.

Additionally, the findings from our review have important policy implications. First, our findings indicate that low employment rates are not directly caused by social housing itself. Second, neither blaming individuals nor simply targeting the environment through housing innovations are sufficient to improve employment outcomes and quality of life for social housing residents. Further research can help elucidate how both personal characteristics and environmental factors influence employment outcomes in the context of social housing, leading to more innovative, multifaceted, holistic, and tailored interventions and support services. Intervention approaches that engage residents, utilise the expertise and positionality of social housing providers/landlords, and incorporate diverse community partnerships can better address the many, often intersecting, personal and environmental barriers and support social housing residents to flourish.

## Figures and Tables

**Table 1:** Inclusion and exclusion criteria.

Inclusion	Exclusion
After 2012. Focus on employment, i.e., outcomes, pathways, barriers, programs. Individuals living in social/public/subsidised housing. Adults over 18 and relevant to working-age adults.	Focus on homelessness or transitional/short term housing e.g., Housing First program. Focus only on housing vouchers or scattered-site rental assistance. Employment is not major outcome/focus or inadequately described. Reviews, theses, books (or chapters), conference proceedings, opinion pieces.

## Data Availability

No new data were created or analysed in this study. Data sharing is not applicable to this article.

## Appendix A

**Table A1:** CINAHL database search 6 December 2022.

#	Query	Results
S22	S10 AND S19	101
S21	S10 AND S19	312
S20	S10 AND S19	463
S19	S11 OR S12 OR S13 OR S14 OR S15 OR S16 OR S17 OR S18	285,357
S18	“affirmative business*”	7
S17	“social firm*”	30
S16	“social enterprise*”	394
S15	““employment program*””	1309
S14	“job”	101,844
S13	(MH “Work+”)	8829
S12	(MH “Employment+”)	53,796
S11	“employ*”	211,913
S10	S1 OR S2 OR S3 OR S4 OR S5 OR S6 OR S7 OR S8 OR S9	6483
S9	“subsidi* housing”	144
S8	“council housing”	22
S7	“low-income housing”	922
S6	“low income housing”	922
S5	“rent geared to income”	2
S4	“rent-geared to income”	2
S3	“affordable housing”	4890
S2	“social housing”	952
S1	(MH “Public Housing”) OR “public housing”	1535
